# Event and Comment

**Published:** 1913-06-15

**Authors:** 


### DENTAL REGISTER.

**Vol. LXVII. June 15, 1913. No. 6**

### EVENT AND COMMENT.

SAMUEL A. CROCKER, Sr. In another place of this issue will be found a detailed statement of the death of Sam'l A. Crocker, Sr., the president of the Company which for many years has published the *Dental Register*. When a man who has occupied so large a place in the affairs of men is called away by death, the loss to the world is not only keenly felt by many warmly attached personal friends, but the loss to the community in which he lived as well as the larger world which he influenced and served must stop and consider his accomplishments. It is not practicable for us to do this in any accurate or satisfactory manner in the case of one who has had so long a career of usefulness and which has extended not only over time but has exerted its influence over many individuals and a widely extended sphere. Who can enumerate the many kind and helpful words of advice which such a man as Mr. Crocker is continually offering to the young men who came to him for help in the beginning of their professional career? Every dental supply man in the country has to meet this problem almost daily with some one, and in just the measure that he meets it with kindness and ability he exerts a tremendous influence on the future of these young men. We shall never forget our interview with Mr. Crocker, when we were considering the most momentous problem that up to that time had come into our life; namely, the purchase of our outfit and venturing on a business career in a great city where we had decided to locate and with no money and no acquaintances except a few patients secured through the College Clinic. His advice was characteristic of the man, it was truthful, but as we thought severe and bluntly put, but yet it was kindly done and with a surprising confidence that completely won our affection and placed upon us a responsibility that challenged our determination to not only succeed for our own good, but to justify the confidence of the man who was willing to trust us. That friendship then begun has never lapsed or been even threatened during all the more than thirty-five years since elapsed, and because our business relations have been more or less constant during this time there should have been many chances for differences of opinion that might have been disastrous to so long a friendship. Our great pleasure now is that we had the great privilege of falling in with such a man in our early career, and that the confidential relations then entered upon have been so sacredly kept by us both. Our friend is not dead to us at all, we rejoice in this precious memory and our spiritual communion will last for this life and beyond. We have little doubt that many men in the profession would make a similar tribute to a man of such sterling character, had they the opportunity; as we know our experience has been that of hundreds of other men. Our friend has gone from us to the great unknown, but as long as we shall live, his life will live with us, a precious memory that will still guide us into stronger and more useful devotion to the great cause of generous service as the best and most profitable achievement in this world.

IMPORTANT DENTAL RECOGNITION. Governor Cox of Ohio, has seen fit to signally honor our profession by appointing a member of it to a place on the State Board of Health, Dr. H. C. Brown of Columbus, has been so appointed. This is probably the first appointment of this kind, although the Governor of Michigan appointed Dr. C. H Oakman, a dentist, a member of the Detroit Board of Health, a few years ago. The significance of all this is not so much that the dentist is getting into politics, but that the public, and thinking people are beginning to recognize the important function of the dentist as conservers of the public health. So long as we have been content to be mere rescurers of the unfortunate from suffering, we have been looked upon as good enough people to have around when needed, and we have been accorded a measure of respect because we have proved our usefulness in the community and attained more than an average amount of culture of the better things. But since we have struck out on a benevolent career, with the idea of preventive dentistry prominently before us and the community, people are beginning to look upon us in a different way, and it is not surprising that Governor Cox has thought it wise, to place a capable dentist on his staff of health preservers. If our attitude is kept progressive we shall have all the honors of any of the older professions, and without seeking them.

PERFECTING THE GOLD INLAY. We are reprinting in this issue a paper printed in the May, *Items of Interest*, on the value of anatomical restorations with the gold inlay, by Dr. J. Lowe Young, and with comments by Dr. Ottolengui. It is a paper that every dentist should read and study carefully, whether he is using the gold inlay in practice or not, as it calls attention to this subject in a way that has not heretofore been definitely presented. It is true that many operators have striven to reach the results here strongly advocated. Two instances have come under our observation during the past year, where the importance of the restoration of the marginal ridges and their complete restoration was strongly advocated by Dr. Bunting, at the Northern Ohio meeting at Sandusky, last June; and Dr. Bates, at the last meeting of the Michigan State Society, presented a set of wax carvers which he had designed for carving the fissures and marginal ridges in the wax patterns. Then our Orthodontists and Prosthodontists have been hammering away at this idea for several years. It is now, however, completely presented by Dr. Young in a positive and convincing statement and as Dr. Ottolengui says—no gold inlay worker from this time, can ignor this important feature of his work and do his whole duty. Read the article carefully and get the idea planted in your mind and you will surely adopt the principle and so refine the technic and perfect your practice.

